# What concentration of tranexamic acid is needed to inhibit fibrinolysis? A systematic review of pharmacodynamics studies

**DOI:** 10.1097/MBC.0000000000000789

**Published:** 2019-01-04

**Authors:** Roberto Picetti, Haleema Shakur-Still, Robert L. Medcalf, Joseph F. Standing, Ian Roberts

**Affiliations:** a Clinical Trials Unit, London School of Hygiene and Tropical Medicine, London, UK; b Australian Centre for Blood Diseases, Central Clinical School, Monash University, Melbourne, Victoria, Australia; c Infection Inflammation and Immunity, UCL Great Ormond Street Institute of Child Health, London, UK

**Keywords:** fibrinolysis, pharmacodynamics, systematic review, tissue plasminogen activator, tranexamic acid

## Abstract

Intravenous tranexamic acid (TXA) reduces death because of bleeding in patients with trauma and postpartum haemorrhage. However, in some settings intravenous injection is not feasible. To find different routes of administration, we first need to determine the minimal concentration of TXA in the blood that is required to inhibit fibrinolysis.

We conducted a systematic review of in-vitro and in-vivo pharmacodynamics studies. We searched MEDLINE, EMBASE, OviSP, and ISI Web of Science from database inception to November 2017 for all in-vitro (including simulated clotting models) or in-vivo studies reporting the relationship between the TXA concentration in blood or plasma and any reliable measure of fibrinolysis.

We found 21 studies of which 20 were *in vitro* and one was *in vivo*. Most in-vitro studies stimulated fibrinolysis with tissue plasminogen activator and measured fibrinolysis using viscoelastic, optical density, or immunological assays. TXA concentrations between 10 and 15 mg/l resulted in substantial inhibition of fibrinolysis, although concentrations between 5 and 10 mg/l were partly inhibitory.

TXA concentrations of 10–15 mg/l may be suitable targets for pharmacokinetic studies, although TXA concentrations above 5 mg/l may also be effective.

## Abstract 2

Supplemental Digital Content is available in the text

## Introduction

Tranexamic acid (TXA) reduces bleeding by inhibiting the enzymatic breakdown of fibrin blood clots (fibrinolysis) [1]. Plasminogen produced by the liver is converted into the fibrinolytic enzyme plasmin by tissue plasminogen activator (tPA). Plasminogen and tPA bind to C-terminal lysine residues on fibrin leading to localized plasmin formation and fibrin cleavage [2]. TXA is a molecular analogue of lysine that inhibits fibrinolysis by competing with fibrin for the lysine-binding sites in plasminogen. TXA inhibits the capacity of plasminogen and plasmin to bind to fibrin, hence preserving blood clots from plasmin-mediated lysis [1].

Intravenous administration of TXA safely reduces death because of bleeding in patients with trauma and postpartum haemorrhage (PPH) [3–5]. In both situations, most deaths occur soon after bleeding onset and treatment delay reduces the survival benefit [6]. It is imperative that patients are treated urgently. TXA is inexpensive, widely available, and heat stable. It is cost effective in sub-Saharan Africa and Southern Asia, where most haemorrhagic deaths occur [7]. Nevertheless, because of long distances to healthcare facilities and a lack of emergency transport many patients are not treated soon enough.

One of the barriers to rapid treatment is the need for an intravenous injection. Health workers able to insert intravenous lines may be unavailable in rural areas and even when they are, securing intravenous access can be difficult in shocked patients with collapsed veins. Although TXA is available for oral and intravenous use, there has been little research into different routes of administration. Intramuscular injection would be easier and would require less training than IV use.

To determine the minimal plasma level of TXA needed to inhibit fibrinolysis, and hence identify whether oral or intramuscular injection would be a viable treatment option, we conducted a systematic review of pharmacodynamics studies. We reviewed in-vivo and in-vitro studies reporting both the concentration of TXA in blood or plasma and its effects on any reliable measure of fibrinolysis. This work will inform future studies into the bioavailability of TXA after oral, intramuscular, and subcutaneous administration.

## Methods

The systematic review methods were published in advance of data collection on the PROSPERO register (protocol number CRD42017079646). The review is reported in accordance with PRISMA guidelines [8].

### Inclusion criteria

Types of studies: In-vitro and in-vivo studies reporting the effects of TXA on fibrinolysis in human blood, plasma, or simulated clotting models.

Methods to measure TXA in the blood and plasma include HPLC, liquid chromatography–mass spectrometry, gas chromatography, or paper electrophoresis [9–11].

Eligible fibrinolysis assays included:

Whole blood tests. These assess fibrinolysis in the presence of platelets and blood cells.

Thromboelastography (TEG) and rotational thromboelastometry (ROTEM) measure the viscoelastic properties of blood during clot formation and fibrinolysis *in vitro*. Comparisons with biochemical assays (e.g., plasmin–antiplasmin complexes) suggest that they may be insensitive to low-grade hyperfibrinolysis [12,13].

The halo assay is a high throughput method that involves forming halo-shaped, tissue factor-induced blood clots in 96 well plates and measuring fibrinolysis using a spectrophotometer [14].

Global fibrinolytic capacity involves measuring fibrin degradation products by ELISA in clotted-lysing blood. Global fibrinolytic capacity is estimated after correcting for background fibrin degradation products in parallel blood samples incubated with the direct plasmin inhibitor, aprotinin. Because no activators are used, endogenous fibrinolysis is assessed [15,16].

Plasma tests: A limitation of these tests is that platelets, cells, and some proteins are absent [14].

The overall fibrinolytic potential and global fibrinolytic potential of plasma is assessed by measuring plasma turbidity in a spectrophotometer after addition of tPA [16]. The initiation of coagulation and subsequent fibrinolysis change the optical density of the plasma. Other optical assays include the clot formation and lysis (CloFAL) assay [16,17] and the clot lysis assay (for example, as described by Niego *et al.*[18]).

ELISA and other immunoassays measure specific fibrinolytic markers such as D-dimer [17,19]. D-dimer can be measured by ELISA (quantitative), whole blood agglutination (qualitative), or latex bead agglutination (quantitative or qualitative). Results from different assays are not comparable and false positives can result from other factors influencing D-dimer levels [17,19].

To facilitate comparisons, concentrations of tPA were converted to ng/ml using the following formula: 10 000 IU/ml = 20 000 ng/ml = 300 nM [20]. Similarly, urokinase-type plasminogen activator (uPA) concentrations were converted to ng/ml with the following formula: 3200 IU/ml = 20 000 ng/ml = 380 nM [21].

### Types of participants

Studies on blood from healthy volunteers or patients. Animal studies were excluded because the concentration needed to inhibit fibrinolysis may be different from humans [22,23]. Animal (e.g., murine) plasma clots are known to be more resistant to lysis with human tPA [24].

### Types of interventions

TXA is added to blood, plasma, or clotting models *in vitro* or given by any route (*in vivo*).

### Types of outcomes

Concentration of TXA (mg/l) needed to reduce fibrinolytic activity to a clinically significant level, for example, 80% reduction in activity [9], or 95% maximal effective concentration (EC95) [25].

### Search

We searched MEDLINE, EMBASE, OviSP, and the ISI Web of Science from the date of database inception to 6 November 2017 using the following terms and keywords:

TXA, amino methyl carboxylic acid, amino methyl cyclohexane acid, cyklokapron, pharmacodynamics, pharmacokinetics, blood, plasma, human, fibrinolysis, fibrin, fibrinogen, antifibrinolytic, antifibrinolysis, clot, lysis, turbidity, immunoassay, immunochemistry, TEG, ROTEM, intravenous, oral, intramuscular, dose, dosing, concentration, and haemorrhage (see Appendix for detailed search strategy). Truncation symbols were used with the text words, when appropriate, to capture variations in spelling and word endings. We also examined the references of systematic reviews located during the search and performed a forward search with the eligible studies to identify further potential articles.

### Study selection

Two reviewers independently assessed records to determine whether they met the inclusion criteria. Titles and abstracts were screened and the full texts of any potentially relevant reports were assessed for inclusion. Two reviewers independently assessed the full texts to confirm eligibility. Disagreements between reviewers were resolved by consensus.

### Data extraction

Data were extracted into a prespecified table including: method to detect TXA in blood or plasma, protocol for TXA administration or addition, fibrinolytic assay used, outcome of fibrinolysis assay, and TXA concentration that achieved a significant reduction of fibrinolysis.

### Data synthesis

The results of each study were described with no mathematical synthesis of results. We examined graphically the relationship between the concentration of tPA used to stimulate fibrinolysis and the minimal inhibitory TXA concentration (80% inhibition of fibrinolysis) and calculated a regression line with 95% confidence intervals (95% CI). The regression was calculated with Stata statistical software: Release 15 (College Station, Texas, USA).

## Results

**Fig. 1:**
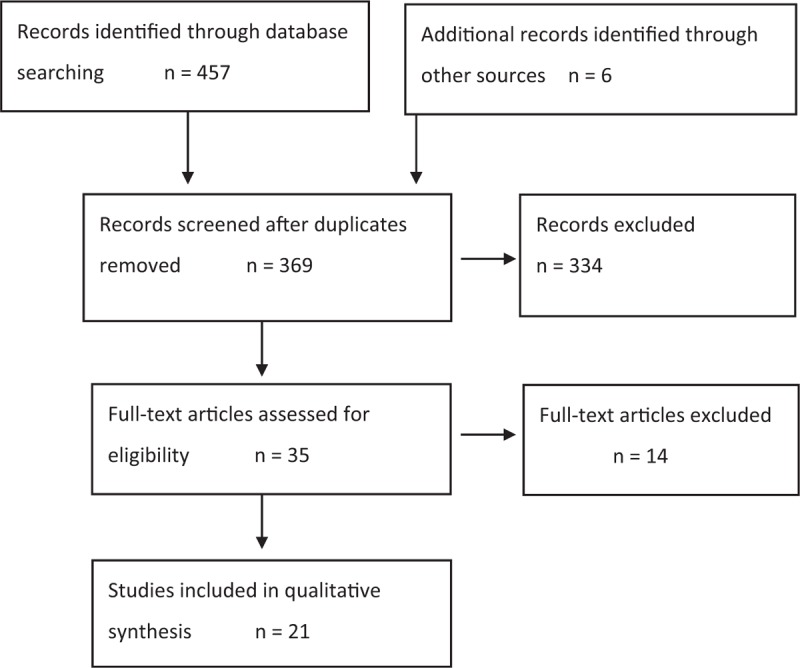
Flow diagram of search. Modified with permission [8].

We found 369 potentially eligible records. After screening titles and abstracts we reviewed the full text of 35 records. We found 21 studies meeting the inclusion criteria (Fig. 1), one of which was unpublished [personal communication]. The first study was published in 1968. All studies were published in English.

The results are shown in Tables 1 and 2. We found 20 in-vitro and one in-vivo study. There were 16 in-vitro studies in which TXA was added to blood or plasma drawn from healthy volunteers or commercial sources, and four studies using clotting models or fibrin plates. One clinical trial measured TXA levels and fibrinolysis in blood from surgical patients.

**Table 1:** Data extracted from in-vitro studies with the minimum effective dose that inhibits 80% or more of fibrinolysis

References	Material	Fibrinolysis activator	TXA doses	Fibrinolysis detection	Fibrinolysis outcome	Minimal TXA effective dose
[9]	Fibrin plates	Homogenates from different human tissues	0–100 mg/l	Lysed area (mm^2^)	Inhibition of tissue activators activity TXA 100 mg/l: 98% reduction TXA 25 mg/l: 90% reduction TXA 10 mg/l: 80% reduction	10 mg/l
[26]	Plasma from 10 inhibitor patients	tPA 400 ng/ml	5, 10 mg/l	Optical density at 405 nm	TXA 0 mg/l: 6 min (clot lysis index) TXA 5 mg/l: 16 min (14.5–17.0) TXA 10 mg/l: 37 min (35.5–38.0) [Median (range of four experiments)]	5 mg/l
[27]	Whole blood and plasma from volunteers	tPA 100 ng/ml in plasma tPA 200 ng/ml in blood	0.09, 0.16, 0.47, 0.94, 1.57, 3.14, 4.72 mg/l	ROTEM (whole blood) Optical density at 405 nm (plasma)	Whole blood: LY60 IC50 = 0.43 mg/l (0.29, 0.51) Plasma: fibrinolysis % IC50 = 0.66 mg/l (0.48, 0.67) [Median (25th; 75th percentile)]	1.57 mg/l (whole blood)
[28]	Blood from 13 healthy volunteers	tPA 100 ng/ml	330 mg/l	ROTEM	TXA 0 mg/l CLI45: 78% (72%/85.5%) CLI60: 21% (7%/59%) TXA 330 mg/l CLI45: 94% (92%/96%) CLI60: 90% (89%/92%) [Median (25th; 75th percentile)]	330 mg/l
[29]	Blood from healthy volunteers	tPA 100 ng/ml	330 mg/l	ROTEM	CLI45, saline: 93% (91/96) CLI45, tPA: 64% (48/80) CLI45, tPA + moderate acidosis: similar to tPA alone CLI45, tPA + moderate acidosis + TXA: 94% (93%/98%) CLI45, tPA + severe acidosis: 49% (26/71) CLI45, tPA + severe acidosis + TXA: 92% (86.5%/94%) [Median (25th; 75th percentile)]	330 mg/l
[30]	Plasma from volunteers	APSAC 7.6 nM tPA 5333 ng/ml	10, 40, 157.2 mg/l	Clot lysis (fibrin) Clauss method (fibrinogen)	TXA 10 mg/l: 80% inhibition of ongoing APSAC-induced fibrinolysis tPA: TXA 10 and 40 mg/l: about 34% inhibition ongoing fibrinogenolysis TXA 157.2 mg/l: inhibition initiation fibrinolysis TXA 40 mg/l: >80% inhibition from the initiation of fibrinolysis for 4 h TXA 10 mg/l: 70% inhibition after 1 h and 30% inhibition after 4 h from the initiation of fibrinolysis	10 mg/l
[22]	Commercial plasma	tPA 2000 ng/ml	2.5, 5, 7.5, 10, 12.5, 15 mg/l	TEG	TXA 12.5 mg/l: 16% EPL TXA 15 mg/l: 0% EPL	14.7 mg/l (95% CI 13.7–15.6)
[31]	Whole blood from healthy volunteers	tPA: 0, 67, 267, 1333 ng/ml	3, 30, 300 mg/l (equivalent to the dose in patients in the CRASH-2 trial)	Clauss method (fibrinogen) TEG	Fibrinogen concentration when blood was spiked with tPA 1333 ng/ml: 1.6 g/l (1.4–1.6) with TXA 0 mg/l 2.2 g/l (2.0–2.3) with TXA 3 mg/l 2.6 g/l (2.3–2.7) with TXA 30 mg/l 2.5 g/l (2.3–3.0) with TXA 300 mg/l [Median (25th; 75th percentile)] tPA 267 ng/ml TXA 3 mg/l only partially corrected TEG parameters TXA 300 mg/l corrected LY30, LY60, EPL, MA, G tPA 1333 ng/ml TXA 300 mg/l normalized only LY30 and EPL	3 mg/l
[32]	Commercial platelet-poor plasma	tPA 167 ng/ml Plasma was mixed with a thrombin inhibitor or a FXa inhibitor	0.4, 9.5 mg/l	Optical density at 405 nm	Plasma + thrombin inhibitor: 80 cycles (1 cycle = 30 s) Plasma + thrombin inhibitor + TXA 0.4: 100 cycles Plasma + thrombin inhibitor + TXA 9.5: no fibrinolysis Plasma + FXa inhibitor: 50 cycles Plasma + FXa inhibitor + TXA 0.4: 100 cycles Plasma + FXa inhibitor + TXA 9.5: no fibrinolysis	9.5 mg/l
[33]	Purified system	tPA 0.5 ng/ml	0–2 mg/l	Fibrin solubilization	TXA 2 mg/l: 50% of fibrin released in 200 min (control 100 min)	2 mg/l
[34]	Whole blood from six healthy volunteers	tPA 225 ng/ml	1, 2, 3 mg/l	TEG	TXA 1 and 2 mg/l inhibited clot lysis in the model of moderate fibrinolysis TXA 3 mg/l: LY30 <8% in model of severe fibrinolysis	1 mg/l (moderate fibrinolysis)
[35]	Commercial pooled plasma	Staphylokinase 30 nM Streptokinase 100 nM	0.16–7.86 mg/l 1.6–78.6 mg/l	Clot lysis	TXA 0.63 mg/l: fibrinolytic potency of Staphylokinase to 50% at 2 h TXA 3.14 mg/l: fibrinolytic potency of Streptokinase to 50% at 2 h Addition of TXA 30 min after Staphylokinase or 15 min after Streptokinase TXA 2.36 mg/l: fibrinolytic potency of Staphylokinase to 50% at 90 min TXA 11 mg/l: fibrinolytic potency of Streptokinase to 50% at 90 min	2.36 mg/l with staphylokinase 11 mg/l with streptokinase
[36]	Purified system	Homogenates from different human tissues	7.86, 15.72, 23.58 mg/l	Optical density at 405 nm	Microtiter plate clot lysis experiments TXA 0 mg/l: 100% lysis rate TXA 7.86 mg/l: 75% lysis rate TXA 15.72 mg/l: 45% lysis rate TXA 23.58 mg/l: 20% lysis rate Inhibition of plasminogen activation TXA 0 mg/l: 100% activation rate TXA 3.93 mg/l: 65% activation rate TXA 7.86 mg/l: 40% activation rate TXA 11.79 mg/l: 30% activation rate TXA 15.72 mg/l: 20% activation rate	15.72 mg/l
Longstaff [unpublished]	Whole blood or plasma	tPA 333 ng/ml uPA 263 ng/ml	1.57, 15.72, 157.21 mg/l	ROTEM Halo assay: optical density	ROTEM: TXA 1.57 mg/l inhibited fibrinolysis Almost complete inhibition in whole blood and plasma (>90%) TXA 40 mg/l with tPA 2 mg/l with uPA and whole blood 3 mg/l with uPA and plasma	1.57 mg/l
[18]	Commercial plasma	tPA 667 ng/ml	1.57, 15.72, 157.21 mg/l	Optical density at 405 nm	Complete lysis block with TXA 15.7 mg/l	15.7 mg/l
[37]	Fibrin plates	Human plasma activator of plasminogen	0.16, 1.6, 15.7 mg/ml	Lysed area (mm^2^)	Inhibition of tissue activators activity: TXA 0.16 mg/l: 50% reduction TXA 1.6 mg/l: 90% reduction TXA 15.7 mg/l: 100% reduction	1.6 mg/ml
[25]	Blood from 10 adult volunteers and 20 children	tPA 3070 ng/ml	2.5, 5, 7.5, 10, 12.5, 15, 17.5, 20, 50, 100 mg/l	ROTEM (LI30)	Adults: EC50 6.8 mg/l (95% CI, 6.4–7.2) EC95 11.3 mg/l (95% CI, 10.6–12.9) Children: EC50 2.8 mg/l (95% CI, 2.6–3.0) EC95 8.6 mg/l (95% CI, 6.9–14.9)	Adults: 11.3 mg/l (95% CI, 10.6–12.9) Children: 8.6 mg/l (95% CI, 6.9–14.9)
[38]	Commercial plasma	tPA 328 ng/ml	0.47–471.63 mg/l	Optical density at 405 nm	IC50 3.79 ± 0.17 mg/l	IC50 3.79 ± 0.17 mg/l
[39]	Plasma from healthy volunteers. Purified system	uPA 1250 and 6250 ng/ml	157.2 mg/l	Fibrin degradation products Fibrinogen degradation products	Complete inhibition of fibrinolysis Complete or partial inhibition of fibrinogenolysis	157.2 mg/l
[40]	Plasma derived from cord blood. Commercial adult pooled normal plasma	tPA 2000 ng/ml	1, 5, 7.5, 10, 12.5, 15, 20 mg/l	TEG (LY30)	Complete prevention of fibrinolysis (LY30 = 0) Neonatal plasma: 6.54 mg/l (95% CI, 5.19–7.91) Adult plasma: 17.5 mg/l (95% CI, 14.59–20.41)	Neonates: 6.54 mg/l (95% CI, 5.19–7.91) Adults: 17.5 mg/l (95% CI, 14.59–20.41)

APSAC, anisoylated lys-plasminogen streptokinase activator complex; CI, confidence intervals; CLI, clot lysis index; EC, effective concentration; EPL, estimated percentage lysis; FXa, factor Xa; ROTEM, rotational thromboelastometry; TEG, thromboelastography; tPA, tissue plasminogen activator; TXA, tranexamic acid; uPA, urokinase-type plasminogen activator.

**Table 2:** Tranexamic acid doses measured in participants’ plasma and detected fibrinolysis markers at different time points

References	Participants	TXA doses	TXA detection	TXA detected	Fibrinolysis detection	Fibrinolysis outcome
[41]	Two TXA treatment groups One control group	Group 1 (continuous): 10 mg/kg bolus + 1 mg/kg/h during surgery + 10 mg/kg on CPB prime (total dose 22.1–25.1 mg/kg), i.v. Group 2 (discontinuous): 10 mg/kg bolus + 10 mg/kg on CPB prime + 10 mg/kg at the end of CPB (total dose 30 mg/kg), i.v. Control group: similar intervention conditions, no TXA administration	Liquid chromatography/mass spectrometry	No difference between the two treatment groups. Mean TXA levels ranging from 28.0 ± 2.3 and 59.9 ± 7.5 mg/l during CPB Last TXA dose: group 1 45.8 ± 8.4 mg/l and group 2 71.0 ± 5.5 mg/l	Immunoassay for D-dimers Fibrinogen immunoassay	D-dimers levels in control group: 282 ± 54 ng/ml (T0, lowest), 2274 ± 474 ng/ml (T3, highest). In T0, groups 1 and 2 had similar D-dimers levels as the control group Group 1: highest level 562 ± 165 ng/ml in T4 (control 2000 ng/ml) Group 2: highest level 393 ± 93 ng/ml in T2 (control 1900 ng/ml), and in T4, D-dimers level decreased to 277 ± 71 ng/ml Fibrinogen concentration in T3 and T4: Control: 2000 mg/l Group 1: 1100 mg/l Group 2: 1400 mg/l

CPB, cardiopulmonary bypass; i.v., intravenous; TXA, tranexamic acid.

### In-vitro studies

In most studies, fibrinolysis was stimulated with tPA (Table 1).

Andersson *et al.*[9] incubated fibrin plates with tissue homogenates containing tPAs and measured the effect of TXA concentrations up to 100 mg/l. Fibrinolysis (lysed area on plate) was reduced by 98% with 100 mg/l TXA, 90% with 25 mg/l TXA and 80% with 10 mg/l TXA.

Dai *et al.*[26] used tPA to stimulate fibrinolysis in plasma from haemophilia patients who had factor VIII antibodies. Clot lysis time was measured using optical methods. Clot lysis time increased from 6 to 16 min with TXA at 5 mg/l and from 6 to 37 min using 10 mg/l.

Dietrich *et al.*[27] stimulated fibrinolysis in blood (tPA at 200 ng/ml) from healthy volunteers and used ROTEM (LY60, defined as percentage decrease in clot amplitude at 60 min after maximal amplitude) to assess the effect of TXA on fibrinolysis. Fifty percentage inhibition [IC50, median (25th; 75th percentile)] was achieved with TXA 0.43 mg/l (0.29, 0.51) and complete inhibition with TXA 1.57 mg/l. The authors stimulated fibrinolysis in plasma (tPA 100 ng/ml) and studied the decrease in optical density 45 min after maximum optical density. Fifty percentage inhibition (IC50) was achieved with TXA 0.66 mg/l (0.48, 0.67). TXA 1.57 mg/l decreased fibrinolysis to less than 10%.

Dirkman *et al.*[28] stimulated fibrinolysis in blood with tPA (100 ng/ml) and evaluated the effect of a single dose of TXA (330 mg/l) corresponding to 2 g/75 kg body weight using ROTEM. TXA reduced clot lysis at 45 and 60 min [clot lysis index (CLI45) and CLI60]. CLI60 (median, 25th/75th percentile) increased from 21% (7%/59%) without TXA to 90% (89%/92%) with TXA. TXA 330 mg/l increased CLI45 from 64% (48/80) to 94% (93/98) in conditions of moderate acidosis and from 49% (26/71) to 92% (86.5/94) in severe acidosis [29].

Fears *et al.*[30] stimulated fibrinolysis in plasma with anisoylated lys-plasminogen streptokinase activator complex or tPA and measured the lysis time of iodine-radiolabelled fibrin clots. TXA at 10 mg/l reduced anisoylated lys-plasminogen streptokinase activator complex -induced fibrinolysis by 80%. TXA at 157.2 mg/l completely inhibited tPA-induced initiation of fibrinolysis. TXA at 40 mg/l caused 80% inhibition at 4 h, TXA at 10 mg/l caused 70% inhibition at 1 h and 30% inhibition at 4 h. TXA at 10 and 40 mg/l inhibited 34% of ongoing tPA-induced fibrinogenolysis.

Fletcher *et al.*[22] used tPA-stimulated plasma and TEG to calculate the estimated percentage lysis 30 min after maximum clot amplitude. The TXA concentration needed to inhibit fibrinolysis was 14.7 mg/l (95% CI 13.7–15.6).

Godier *et al.*[31] spiked blood samples with 67 ng/ml, 267 ng/ml, and 1333 ng/ml tPA and studied the effect of TXA on fibrinolysis using TEG. 67 ng/ml tPA did not affect the TEG traces but there was a significant effect with 267 and 1333 ng/ml. With 267 ng/ml tPA, a concentration of 3 mg/l TXA partially corrected TEG parameters, whereas 300 mg/l TXA corrected most parameters. With 1333 ng/ml tPA, a concentration of 3 mg/l TXA was ineffective, but 300 mg/l TXA corrected LY60 and estimated percentage lysis. The effect of 30 mg/l TXA was similar to that of 300 mg/l TXA at all tPA concentrations (Table 1). TXA also inhibited fibrinogenolysis (based on the drop in fibrinogen level after tPA addition). Addition of 67 ng/ml tPA did not cause fibrinogenolysis. However, tPA resulted in a concentration-dependent fall in fibrinogen levels at 267 and 1333 ng/ml tPA. This fall was partly prevented by the addition of 3 mg/l TXA and almost fully prevented at a concentration of 30 mg/l or higher.

He *et al.*[32] spiked normal commercial plasma with tPA in the presence of a thrombin inhibitor or a factor Xa inhibitor to mimic an antithrombotic therapy. TXA at 0.4 mg/l prolonged the clot lysis time from 40 to 50 min with a thrombin inhibitor and from 25 to 50 min with a factor Xa inhibitor. TXA at 9.5 mg/l abolished fibrinolysis in both cases.

Hoylaerts [33] used tPA in an in-vitro system where wells of tissue culture plates were coated with iodine-radiolabelled fibrin. Plasminogen was preincubated with several TXA concentrations and added to the wells. The radioactivity released into the solution was the product of the solubilization of fibrin and was measured for up to 200 min. The time required to solubilize 50% of fibrin was calculated. The solubilization time increased from 100 min in the controls to 200 min with 2 mg/l of TXA.

Kostousov *et al.*[34] used tPA to induce moderate fibrinolysis in blood diluted with Ringer's lactate (Hospira, Lake Forest, Illinois, USA) and severe fibrinolysis in blood diluted with Ringer's lactate and Voluven (Hospira, Lake Forest, Illinois, USA). In moderate fibrinolysis, TXA at 1 and 2 mg/l inhibited TEG clot lysis. In severe fibrinolysis, TXA 3 mg/l decreased LY30 to less than 8%.

Lijnen *et al.*[35] induced fibrinolysis in plasma with staphylokinase 30 nM or streptokinase 100 nM. Lysis time of iodine-radiolabelled fibrin clots was measured. The concentrations of TXA needed to reduce clot lysis to 50% of the value measured without TXA after 2 h were 0.63 mg/l with staphylokinase and 3.14 mg/l with streptokinase. In other experiments, TXA was added 30 min after the addition of staphylokinase or 15 min after streptokinase. TXA needed to reduce fibrinolysis to 50% after 90 min was 2.36 mg/l with staphylokinase and 11 mg/l with streptokinase.

Longstaff [36] prepared fibrin clots by mixing fibrinogen and α-thrombin. Plasmin was added when maximal optical density was reached and clot lysis measured for 5 h. Lysis decreased with increasing TXA concentration (Table 1). Plasminogen activation was also assessed in a solution with fibrinogen, thrombin, plasminogen, and tPA. Plasminogen activation dropped to 40% with 7.86 mg/l TXA and 20% with 15.72 mg/l of TXA. TXA between 8 and 15 mg/l reduced clot lysis (75 and 45% lysis rate, respectively) and inhibited plasminogen activation (40 and 20% activation rate, respectively).

Longstaff [unpublished] used ROTEM (maximum clot firmness, clot lysis index) to measure tPA and uPA-induced fibrinolysis. TXA 1.57 mg/l inhibited fibrinolysis almost as efficiently as TXA 157.21 mg/l with both tPA and uPA. Longstaff also used the halo assay [14] with whole blood or plasma. In both cases, 40 mg/l of TXA almost completely inhibited tPA-induced clot lysis and a concentration almost an order of magnitude lower inhibited uPA-induced lysis (Table 1).

Niego *et al.*[18] stimulated fibrinolysis in commercial human plasma with tPA and measured clot turbidity for 100 min. TXA at 15.7 mg/l completely inhibited fibrinolysis.

Okamoto *et al.*[37] incubated fibrin plates and tested the inhibitory activity of TXA on fibrinolysis caused by the human plasma activator of plasminogen. They showed that 0.16 mg/l of TXA inhibited 50% of fibrinolysis, 1.6 mg/l inhibited about 90% of fibrinolysis, and 15.7 mg/l inhibited fibrinolysis almost completely.

Rozen *et al.*[25] used ROTEM to study the effect of TXA on fibrinolysis in blood from children (aged 1–10 years) undergoing cardiac catheterization for congenital heart disease (10 cyanotic and 10 noncyanotic) and blood from healthy adult volunteers. Fibrinolysis was induced with tPA and the effect of TXA on lysis index at 30 min was assessed. The efficacy concentration 50% (EC50) in adults was 6.8 mg/l (95% CI, 6.4–7.2), and the EC95 was 11.3 mg/l (95%CI, 10.6–12.9). In children, EC50 was 2.8 mg/l (95%CI, 2.6–3.0) and EC95 was 8.6 mg/l (95%CI, 6.9–14.9).

Sperzel and Huetter [38] spiked plasma with tPA and assessed fibrinolysis by measuring plasma turbidity in a spectrophotometer following the addition of tPA. TXA concentrations ranged from 0.47 to 471.63 mg/l. TXA inhibited fibrinolysis with an IC50 of 3.79 ± 0.17 mg/l.

Takada *et al.*[39] added either 1250 or 6250 ng/ml of urokinase to plasma and assessed a single dose of TXA (157.2 mg/l) by measuring fibrin and fibrinogen degradation products. For both concentrations of urokinase, TXA inhibited fibrinolysis and fibrinogenolysis. TXA was also tested in a system with plasminogen, thrombin, fibrinogen, and urokinase (6250 ng/ml). TXA completely inhibited fibrinolysis and reduced fibrinogenolysis by about 50%.

Yee *et al.*[40] added tPA to adult plasma and plasma from cord blood collected at caesarean deliveries of 23 full-term (38–42 weeks) singleton live births. The effect of TXA on fibrinolysis was determined with TEG (LY30). The minimum dose of TXA needed to completely inhibit fibrinolysis was 6.54 mg/l (95% CI, 5.19–7.91) in neonatal plasma and 17.5 mg/l (95% CI, 14.59–20.41) in adult plasma.

**Fig. 2:**
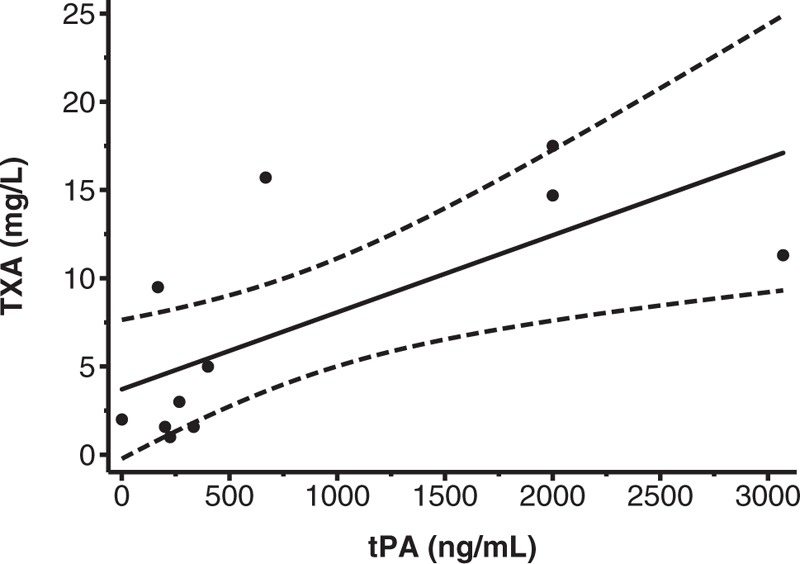
Plot showing the tPA concentrations used in in-vitro studies and the corresponding minimal TXA concentration needed to inhibit at least 80% of fibrinolysis. The regression line with 95% CI is also shown. Five studies were excluded: three studies that used only one very high concentration of TXA to inhibit fibrinolysis [28,29,39], one study that used a concentration of tPA so high [30] that it falls in the range of tPA levels used in thrombolytic treatment (960–1830 ng/ml [52]), and one study that did not report the concentration needed for 80% inhibition of fibrinolysis were excluded [38]. tPA, tissue plasminogen activator; TXA, tranexamic acid.

Figure 2 shows the relationship between the tPA concentration used to induce fibrinolysis and the minimal concentration of TXA needed to inhibit at least 80% of fibrinolysis. The TXA concentration required increased by 0.004 mg/l for every 1 ng/ml of tPA used to stimulate fibrinolysis (95% CI 0.001–0.008, *R*^2^ = 47.26%).

### In-vivo treatments

Couturier *et al.*[41] compared the effect of two TXA dosing schemes with a no TXA group in children undergoing surgery with cardiopulmonary bypass (CPB) (Table 2). The dosing schemes included a continuous administration of TXA (10 mg/kg bolus dose followed with a continuous 1 mg/kg/h infusion until completion of surgery, and then 10 mg/kg dose in the CPB prime), and a discontinuous administration (10 mg/kg bolus dose, then a 10 mg/kg dose in the CPB prime, and 10 mg/kg dose at the end of CPB). They measured TXA, D-dimer, and fibrinogen. TXA levels ranged between 28.0 ± 2.3 and 59.9 ± 7.5 mg/l (mean ± SEM) during surgery. TXA inhibited the bypass-induced D-dimer increase which peaked at 393 ± 93 ng/ml in the discontinuous group and 562 ± 165 ng/ml in the continuous group compared with the no TXA group (2274 ± 474 ng/ml).

## Discussion

Although TXA may have other biological effects, this systematic review aimed to determine the minimum concentration of TXA necessary to inhibit fibrinolysis [42,43]. Our results suggest that TXA concentrations between 10 and 15 mg/l provide near maximal inhibition of fibrinolysis, although concentrations between 5 and 10 mg/l provide significant inhibition (e.g., 18, 32). The minimum TXA concentration might be lower in children [44].

Despite an extensive search for all eligible studies, published or unpublished, it is possible that some studies were missed. Although less is known about the impact of publication bias in systematic reviews of pharmacodynamics studies, than in reviews of clinical trials, selective reporting has been documented and is a potential threat to validity [45,46]. Furthermore, many pharmacodynamics studies fail to report aspects of study methodology (e.g., blinding) that may have a bearing on the results. Because the rate of fibrinolysis in normal blood or plasma is slow, fibrinolytic activators are added to speed up the process. Although differences in the potency of fibrinolytic activators make direct comparisons difficult, our results suggest that the tPA concentration used to stimulate fibrinolysis influences the results. Indeed, the wide range of tPA concentrations used may explain the heterogeneity in inhibitory concentrations. Another potential limitation is the diversity of the methods used to measure fibrinolysis. The validation and standardization of methods to measure fibrinolysis has progressed more slowly than for coagulation testing and widely accepted assays are unavailable.

Can results from in-vitro studies be used to guide the target TXA concentration for patients with trauma and PPH? Cohort studies in patients with major trauma show that plasma tPA concentrations are highest in patients with hypotension and acidosis. Nevertheless, the tPA concentrations seen in even the most severely injured patients are lower than those used to stimulate fibrinolysis *in vitro.* Indeed, tPA levels in trauma patients are generally below 100 ng/ml [47–50]. This suggests that results from in-vitro studies may overestimate the concentration of TXA needed to inhibit fibrinolysis *in vivo*.

In most of the included in-vitro studies, TXA was added either before tPA was added or at the same time. The experimental protocol therefore differs from clinical situations. Only one publication [30] reported the effect of TXA on ongoing fibrinolysis, adding TXA 15–30 min after stimulating fibrinolysis, but very high doses of fibrinolysis activators were used (e.g., 80 nM of tPA, corresponding roughly to more than 5000 ng/ml). For this reason, the extent to which in-vitro results can be applied to the clinical practice is open to question.

Urokinase is a fibrinolytic activator sometimes used in in-vitro experiments. However, clinical studies show that tPA is involved in the early activation of fibrinolysis with levels reaching a peak at about 3 h after injury. uPA levels increase after tPA levels begin to decrease [51]. It is unlikely that uPA plays a major role in fibrinolysis during the early stage of trauma or PPH, when TXA is known to be life saving.

In conclusion, this systematic review suggests that TXA concentrations in the range of 10–15 mg/l result in substantial inhibition of fibrinolysis. These results will inform pharmacokinetic studies to examine the bioavailability of TXA after oral, intramuscular, and subcutaneous administration. If therapeutic levels of TXA can be achieved in a timely manner via these alternative routes, this could have important implications for the prehospital management of patients with trauma and postpartum haemorrhage.

## Acknowledgements

We thank Ms Sarah Dawson, Senior Research Associate in Information Retrieval at the Bristol Medical School (University of Bristol, UK), for designing and performing the literature search. We thank Dr Stanislas Grassin-Delyle and Dr Colin Longstaff for their helpful comments on the manuscript and thank Dr Colin Longstaff for sharing unpublished data.

This study was funded by Wellcome (grant number WT208870/Z/17/Z) and the Bill & Melinda Gates Foundation (grant number OPP1176150) as part of the WOMAN-2 program.

Role of the sponsor: Wellcome and the Bill & Melinda Gates Foundation did not play a role in the design and conduct of the study, collection, analysis, and interpretation of the data, preparation, review, or approval of the manuscript.

### Conflicts of interest

There are no conflicts of interest.

## Supplementary Material

Supplemental Digital Content
