# Supplementary material for: What concentration of tranexamic acid is needed to inhibit fibrinolysis? A systematic review of pharmacodynamics studies
Source: Blood Coagul Fibrinolysis. 2019 Jan 4;30(1):1–10. doi: 10.1097/MBC.0000000000000789 (PMC6365258; doi:10.1097/MBC.0000000000000789)
Supplement: Supplemental Digital Content [file blcof-30-1-s001.pdf]

What concentration of tranexamic acid is needed to inhibit fibrinolysis?
A systematic review of pharmacodynamics studies.

Database search strategies

1. Ovid MEDLINE(R) Epub Ahead of Print, In-Process & Other Non-Indexed Citations, Ovid MEDLINE(R) Daily and Ovid MEDLINE(R) <1946 to 6-Nov-2017>
Search Strategy:
----
1 Tranexamic Acid/pk (35)
2 Tranexamic Acid/ad, pd and *Fibrinolysis/de (66)
3 or/1-2 (101)
4 Tranexamic Acid/ (2880)
5 (Tranexamic or TXA or AMCA or AMCHA or cyklokapron).ti,ab,kf,rn. (5357)
6 or/4-5 (5357)
7 (fibrinolysis or fibrinolytic or hyperfibrin* or hypofibrin*).ti,ab,kf,hw. (66206)
8 (fibrin* adj3 (activat* or activity or bind* or challenge or degrad* or inhibit* or reduc*)).ti,ab,kf. (23874)
9 (CLT or (clot adj (formation or breakdown or degrad* or inhibit* or lysis or reduc*))).ti,ab,kf. (6266)
10 Fibrin Fibrinogen Degradation Products/ (8184)
11 (FDP or (fibrin* adj3 degrad*) or D-Dimer*).ti,ab,kf. (15135)
12 (coagulation potential or coagulability).ti,ab,kf. (1229)
13 or/7-12 (90222)
14 blood coagulation tests/ (19255)
15 (((blood or plasma or laboratory or point-of-care) adj2 test) or assay or immunoassay or ELISA).ti,ab,kf. (778158)
16 exp Immunoassay/ (501474)
17 Thrombelastography/ (4794)
18 (thromboelastogra* or TEG or thromboelastomet* or ROTEM or halo assay).ti,ab,kf. (4592)
19 (h?emostasis potential or OHP or turbidity or global fibrinolytic capacity or GFC).ti,ab,kf. (11524)
20 (fibrin* adj3 (in-vivo or in-vitro or ex-vivo)).ti,ab,kf. (1632)
21 or/14-20 (1163149)
22 Dose-Response Relationship, Drug/ (401740)
23 (dose? or megadose? or dosing).ti,ab,kf. (1310518)
24 (pharmacokinetic? or pharmacodynamic? or drug kinetic? or bioavailability).ti,ab,kf,hw. (213933)
25 ((Tranexamic or TXA) adj3 concentration?).ti,ab,kf. (71)
26 ((blood or plasma or exogenous) adj5 (concentration? or availability)).ti,ab,kf. (240750)
27 Tranexamic Acid/an, bl [Analysis, Blood] (52)
28 or/22-27 (1796102)
29 6 and 13 and (21 or 28) (384)
30 (administ* or inject? or route or intraveneous or oral* or ingestion or sublingual or intraosseous or intramuscular or subcutaneous or transdermal).ti,ab,kf. (1985961)
31 (healthy or volunteer?).ti,ab,kf. (813315)
32 phase 1 study.ti,ab,kf. (1244)
33 6 and 30 and (31 or 32) (50)
34 (screen* or detect*).ti,ab,kf,hw. (2697692)

35 (precise or precision or accurate or accuracy or reproducibility or reproducible or sensitiv* or specific* or validat* or validity or reliable or reliability or standard*).ti,ab,kf. (5595781)
36 "Reproducibility of Results"/ (371195)
37 exp "Sensitivity and Specificity"/ (547690)
38 or/34-37 (7404647)
39 29 and 38 (97)
40 3 or 33 or 39 (235)
41 (exp Animals/ or Animal Experimentation/ or exp Models, Animal/) not (exp Human Experimentation/ or Humans/) (4687080)
42 ((rat or rats or rodent* or mouse or mice or murine or rabbit or rabbits or pigs or piglets or swine or porcine or sheep or lambs or ovine or cat or cats or feline or dog or dogs or canine or horse? or equine or cattle or bovine or monkey or marmoset?) not (human? or man)).ti. (2012897)
43 40 not (41 or 42) (195)

****

2. Ovid Embase <1974 to 2017 Week 45>
Search Strategy:
----
1 ((Tranexamic or TXA) adj3 concentration?).ti,ab,kw. (101)
2 tranexamic acid/cr, do, pk, pd [Drug Concentration, Drug Dose, Pharmacokinetics, Pharmacology] (1144)
3 *fibrinolysis/ or fibrinolysis.ti. (19020)
4 (1 or 2) and 3 (84)
5 tranexamic acid/ (10318)
6 (Tranexamic or TXA or AMCA or AMCHA or cyklokapron).ti,ab,kw,rn. (11350)
7 5 or 6 (11382)
8 (fibrinolysis or fibrinolytic or hyperfibrin* or hypofibrin*).ti,ab,kw,hw. (86736)
9 (fibrin* adj3 (activat* or activity or bind* or challenge or degrad* or inhibit* or reduc*)).ti,ab,kw. (28006)
10 FDP.ab. (3248)
11 fibrin degradation product/ (3695)
12 D dimer/ (15932)
13 d-dimer?.ti,ab,kw. (14309)
14 (coagulation potential or coagulability).ti,ab,kw,hw. (1411)
15 or/8-14 (116234)
16 blood examination/ (12265)
17 blood clotting test/ (13175)
18 ((blood or plasma or laboratory) adj2 test?).ti,ab,kw. (84929)
19 laboratory test/ or exp immunoassay/ or enzyme linked immunosorbent assay/ (633832)
20 "point of care testing"/ (9694)
21 (assay or immunoassay or ELISA).ti,ab,kw,hw. (1301901)
22 thromboelastography/ (7157)
23 (thromboelastogra* or TEG or thromboelastomet* or ROTEM or halo assay).ti,ab,kw. (6955)
24 (h?emostasis potential or OHP or turbidity or global fibrinolytic capacity or GFC).ti,ab,kw. (14250)

25 (in-vivo or in-vitro or ex-vivo).ti,ab,kw,hw. (2534364)
26 or/16-25 (3820753)
27 concentration response/ or dose response/ or *drug response/ (511691)
28 exp *pharmacokinetic parameters/ (40600)
29 pharmacokinetic parameters/ or maximum plasma concentration/ or minimum plasma concentration/ or time to maximum plasma concentration/ (41263)
30 drug blood level/ or drug concentration/ (214800)
31 dosage schedule comparison/ or drug dose/ (31666)
32 (dose or megadose or dosing).ti,ab,kw,hw. (1906512)
33 exp pharmacokinetics/ (636262)
34 exp pharmacodynamics/ (3308492)
35 (pharmacokinetic? or pharmacodynamic? or drug kinetic? or bioavailability).ti,ab,kw. (271093)
36 ((blood or plasma or exogenous) adj5 (concentration? or availability)).ti,ab,kw. (273308)
37 (concentration? or bioavailability).ti. (183268)
38 or/27-37 (5007432)
39 7 and 15 and 26 and 38 (277)
40 diagnostic accuracy/ (222438)
41 "sensitivity and specificity"/ (287582)
42 exp reliability/ (147198)
43 (screen* or detect*).ti,ab,kw,hw. (3489147)
44 (precise or precision or accurate or accuracy or reproducibility or reproducible or sensitiv* or specific* or validat* or validity or reliable or reliability or standard*).ti,ab,kw. (6656267)
45 Gold standard/ (42694)
46 consensus/ (50989)
47 validity/ (44067)
48 screening/ or screening test/ (230406)
49 intermethod comparison/ (231748)
50 practice guideline/ (322403)
51 or/40-50 (9183650)
52 39 and 51 (104)
53 phase 1 clinical trial/ (40303)
54 normal human/ (730820)
55 (healthy or volunteer?).ti,ab,kw. (1035912)
56 or/53-55 (1574780)
57 ((Tranexamic or TXA or AMCA or AMCHA or cyklokapron) adj3 (administ* or inject? or route or intraveneous or oral* or ingestion or sublingual or intraosseous or intramuscular or subcutaneous or transdermal)).ti,ab,kw. (919)
58 56 and 57 (33)
59 Animal experiment/ not (human experiment/ or human/) (1947395)
60 ((rat or rats or rodent* or mouse or mice or murine or rabbit or rabbits or pigs or piglets or swine or porcine or sheep or lambs or ovine or cat or cats or feline or dog or dogs or canine or horse? or equine or cattle or bovine or monkey or marmoset?) not (human? or man)).ti. (2155370)
61 59 or 60 (3191461)
62 (4 or 52 or 58) not 61 (184)

****

3. Web of Science (6-Nov-2017)
Indexes=SCI-EXPANDED, CPCI-S, ESCI Timespan=1900-6-Nov-2017
#9 (#6 or #7 or #8) (n=78)
#8 TOPIC: ((Tranexamic SAME (healthy and (volunteer* or subject*)))) AND TOPIC: ((concentration* or bioavailability or pharmacokinetic* or pharmacodynamic*))
#7 TI=(Tranexamic or TXA or AMCA or AMCHA or cyklokapron) AND "healthy volunteer*" AND TI= (administ* or inject* or route or intraveneous or oral* or ingestion or sublingual or intraosseous or intramuscular or subcutaneous or transdermal or dose or megadose or dosing)
#6 (#1 and #2 and #3 and #4 and #5)
#5 TOPIC: (diagnos* or screen* or detect* or precise or precision or accurate or accuracy or reproducibility or reproducible or sensitiv* or specific* or validat* or validity or reliable or reliability or standard* or consensus or comparison)
#4 TOPIC: ("concentration response" or "dose response" or "drug response" or "plasma concentration" or "blood level" or "drug concentration" or "dosage schedule" or dose or megadose or dosing or pharmacokinetic* or pharmacodynamic* or "drug kinetic*" or bioavailability) or TOPIC: ((blood or plasma or exogenous) same (concentration? or availability)) OR TITLE: (concentration*)
#3 TOPIC:((blood or plasma or laboratory) same test*) or TOPIC:(assay or immunoassay or ELISA or thromboelastogra* or TEG or thromboelastomet* or ROTEM or "halo assay" or "hemostasis potential" or "haemostasis potential" or OHP or turbidity or "global fibrinolytic capacity" or GFC or in-vivo or in-vitro or ex-vivo)
#2 TOPIC: (fibrinolysis or fibrinolytic or hyperfibrin* or hypofibrin* or TOPIC: (fibrin* same (activat* or activity or bind* or challenge or degrad* or inhibit* or reduc*)) or TOPIC: (FDP or d-dimer* or "coagulation potential" or coagulability)
#1 TOPIC: (Tranexamic or TXA or AMCA or AMCHA or cyklokapron)

****
